# The fate of cellulose nanocrystal stabilised emulsions after simulated gastrointestinal digestion and exposure to intestinal mucosa†
†Electronic supplementary information (ESI) available. See DOI: 10.1039/c8nr05860a


**DOI:** 10.1039/c8nr05860a

**Published:** 2019-01-30

**Authors:** Alan Mackie, Simon Gourcy, Neil Rigby, Jonathan Moffat, Isabel Capron, Balazs Bajka

**Affiliations:** a School of Food Science and Nutrition , University of Leeds , Leeds , LS2 9JT , UK . Email: a.r.mackie@leeds.ac.uk; b Univ Angers , Inst Univ Technol , F-49016 Angers , France; c Institute of Food Research , Norwich Research Park , Norwich , NR47UA , UK; d Asylum Research , an Oxford Instruments Company , High Wycombe , HP12 3SE , UK; e INRA , Biopolymeres Interact Assemblages UR1268 , F-44316 Nantes , France; f Department of Nutritional Sciences , King's College London , London , SE1 9NH , UK

## Abstract

It is well recognised that the average UK diet does not contain sufficient fibre. However, the introduction of fibre is often at the detriment of the organoleptic properties of a food. In this study on the gastrointestinal fate of nanoparticles, we have used cellulose nano-crystals (CNCs) as Pickering stabilising agents in oil in water emulsions. These emulsions were found to be highly stable against coalescence. The CNC and control emulsions were then exposed to simulated upper gastrointestinal tract digestion and the results compared to those obtained from a conventional protein stabilised emulsion. Finally the digested emulsions were exposed to murine intestinal mucosa and lipid and bile absorption was monitored. Importantly, the results show that the CNCs were entrapped in the intestinal mucus layer and failed to reach the underlying epithelium. This entrapment may also have led to the reduced absorption of saturated lipids from the CNC stabilised emulsion *versus* the control emulsion. The results show the potential of CNCs as a safe and effective emulsifier.

## Abstract 2

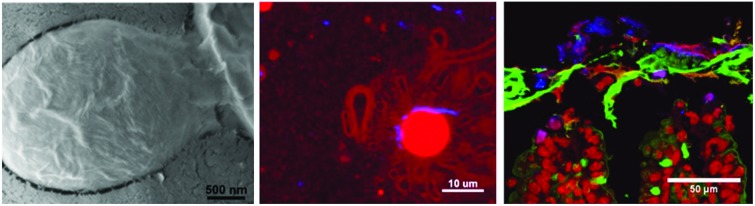
The intestinal mucus layer prevents cellulose nanocrystals from reaching the epithelium and can modulate lipid and bile absorption.

## Introduction

There has been much debate recently about the use of nanoparticles in food.^1^ The standard definition of a nanoparticle is an artificially produced particle that is less than 100 nm in any one dimension. By this definition many food materials already contain nanoparticles.^2^ Indeed a number of researchers have been active in developing nanoscale systems for the enhanced delivery of bioactives.^3^–^5^ Despite this activity there are still some concerns about the safety of nanoscale structures and uncertainty about their fate once consumed.^6^ The gastrointestinal tract is covered by a protective layer of mucus, a complex network of highly branched glycoproteins and other macromolecules such as DNA. The importance of this mucosal layer as a barrier to nanoparticle uptake has been demonstrated previously.^7^ Absorption of particulate material from the small intestine occurs primarily *via* transcytosis in the M-cells of the Peyer's Patches in the gut-associated lymphoid tissue (GALT), and has been described for particles in the range 20–500 nm. Absorption occurs to a lesser extent in enterocytes through clathrin- or caveolin-mediated endocytosis, pinocytosis and phagocytosis.^8^

The use of particles to stabilise emulsion droplets has been known for a long time and was initially described by Pickering.^9^ As a result, such colloidal systems have become known as Pickering emulsions. They have a number of advantages over conventional emulsions associated with high stability against coalescence. This is in part because of the high energy associated with displacing the particles from the interface.^10^ For large particles, the energy may be of the order of 1000 kT depending on the conditions. This is in contrast small molecule surfactants where the energy required may only be a few kT. In addition to the high energy required to remove them from the interface, the surface properties of the particles can dictate the type of emulsion formed. Thus, for largely hydrophilic particles where the contact angle (*θ*) at the interface is small (*θ* < 90°) the particles drive the formation of oil in water emulsions. The large thickness of the particle coating then provides robust steric stabilisation against coalescence. In the food arena there are also a number of Pickering stabilised emulsion and foam examples where the particles may be fat crystals or protein aggregates.^2^ In each case, the properties of the particles determine the properties of the colloid.

Cellulose nanocrystals (CNCs) have been studied since the 1950s but have recently regained interest as a Pickering stabilising agent.^11^ Cellulose is very widely available from many different sources and can be milled or otherwise processed into nanoparticles with relative ease. The use of rods as Pickering agents has been studied theoretically,^12^ showing the importance of the aspect ratio of the rods and the size of the emulsion droplets in the precise form of the 2-D structures formed by the rods. This has implications for tuneable delivery systems. Indeed, there are many examples in the literature of CNCs used to stabilise emulsions.^13^,^14^

Although colloidal systems are widely used in the food industry because of their extremely acceptable organoleptic properties, they have also been associated with highly processed food. This represents a challenge due to the effectiveness of such multi-phase systems ability deliver nutrients to the body. As a result, there is now interest in assessing ways of making such systems healthier, such as the addition of dietary fibre. Soluble fibres are normally considered the most effective as they can delay nutrient absorption through a number of different mechanisms. However, because of the complex nature of the digestive process, there are several other systems that have potential. For example, the use of dietary fibre is one tool that can be used to lower risk factors for cardiovascular disease and type 2 diabetes mellitus.^15^ Several studies have shown efficacy for a range of different dietary fibres. For example, the cereal dietary fibre β-glucan has been shown to lower cholesterol. Although the precise mechanism is not known, it is thought to involve the sequestering of bile acids, probably through entrapment of mixed micelles.

The aim of this study was to determine the fate of cellulose nanoparticles in the upper GI tract. For this purpose we formed cellulose nano-crystal (CNC) stabilised Pickering emulsions and exposed them to simulated upper GI tract digestion. This was followed by exposure to murine intestinal tissue where the absorption of both free fatty acids and bile acids was determined. Using this approach, we aimed to assess the fate of the CNCs following ingestion and measure the effect of the cellulose on the absorption of bile and lipid.

## Experimental

### Materials and methods

**Fig. 1:**
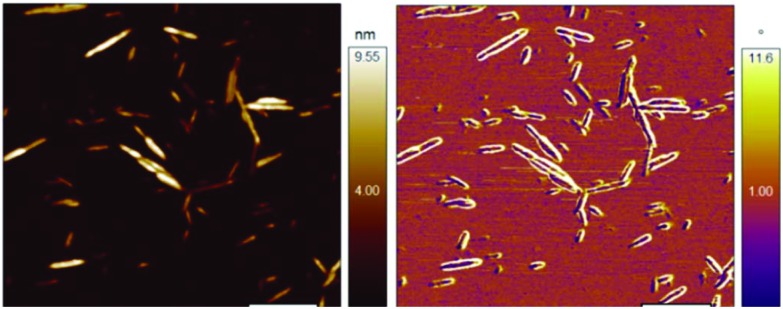
An AFM measurement, topography (left) and phase (right), of typical CNC's deposited from aqueous solution onto mica and imaged under air.

The CNCs were kindly provided by Dr Isabel Capron. Their method of production has been well described previously.^16^ The stock solution of CNCs was 5 mg mL^–1^ in water. The CNCs carry a charge density around 0.2 e nm^–2^ determined by titration. The mean size of the CNCs was determined using a number of different methods. Firstly, the mean (*z*-average) size given by dynamic light scattering (DLS) was 102 nm and was determined using a Malvern Zetasizer Nano-ZS (Malvern Instruments, UK). In addition, atomic force microscopy (AFM) was used to determine the size and shape of the CNCs and a typical image is shown in Fig. 1. Analysis of this and similar images yielded a value of 133 ± 54 nm for the length, 28.9 ± 7.6 nm for the average width and 6–10 nm for the thickness of the rods.

### Emulsion formation

An emulsion was made comprising 10% sunflower oil and 90% 50 mM NaCl containing 1 mg ml^–1^ of CNCs. 1 mL of this mixture was then emulsified using a sonicator with a 3 mm probe tip set to 20% amplitude and a 90% duty cycle for 4 one second pulses. The emulsion was then immediately cooled to 4 °C. The emulsion was found to be highly stable with a means size D(3,2) of 4.0 ± 1.3 μm. The emulsion droplet size distribution was determined using a LS-230 particle sizer (Beckman Coulter Ltd, High Wycombe, UK). For the digestion experiments, 5 mL of emulsion was produced in the same way. A control emulsion using 3.0 mg mL^–1^ sodium caseinate solution in 150 mM NaCl at pH 6.5 was prepared under the same conditions to serve as a control. The size distribution of this emulsion was found to be monomodal, with a D(3,2) of 3.0 ± 0.07 μm.

### Simulated digestion

The CNC and control emulsions were digested using the InfoGest method described by Minekus *et al.*^17^ Briefly, this involved a 2 min oral digestion, then 120 min gastric digestion followed by 120 min of small intestinal digestion.

### Atomic force microscopy

The CNCs were prepared for atomic force microscopy (AFM) by placing a drop of 0.5 mg mL^–1^ CNC solution on a piece of freshly cleaved mica. The drop was left for five minutes before the surface was rinsed with distilled water. Atomic force microscopy measurements were carried out on the Cypher S (Oxford Instruments Asylum Research, Santa Barbara, USA) in amplitude modulated ac mode (commonly known as tapping mode). The probe used was an AC160TS cantilever from Olympus, Japan.

### Confocal microscopy

The CNC stabilised Pickering emulsions were imaged following production, post gastric and post duodenal stages of digestion. Briefly 100 μL sample was incubated with 4 μM Nile Red to stain the lipid and 100 μM Calcofluor to stain the CNC's (Sigma-Aldrich, Poole, UK) for 30 min. 10 μL of the stained samples were transferred to slides, cover-slipped and sealed. Samples were imaged using a Leica SP5 II with Calcofluor excitation at 405 nm and emission between 415–470 nm and Nile red excitation at 488 nm and emission between 550–640 nm. Formalin fixed tissue sampled from the Ussing chamber samples were embedded in paraffin and 4 μm sections were cut onto poly-l-lysine coated slides. Sections were incubated with Oregon green-conjugated wheat germ agglutinin (10 μg mL^–1^) and ToPro-3 (10 μM) for 30 min to stain for mucins/cell membrane glycoproteins and cell nuclei respectively. Samples were then washed in PBS + 0.01% Tween 60, followed by 10 min incubation in Calcofluor (20 μg ml^–1^) to stain the CNC's. Slides were mounted using Fluoroshield media (Sigma, Poole, UK) Samples were imaged using a Leica SP5 II with Calcofluor excitation at 405 nm and emission between 415–470 nm, wheat germ agglutinin (Thermo-Fisher Scientific) excitation at 488 nm and emission between 500–550 nm and ToPro-3 (Thermo-Fisher Scientific) excitation at 633 nm and emission between 640–750 nm. Micrographs and 3D renderings were compiled using ImagePro-plus v7.1 or Fiji (ImageJ v1.51 g).

### Cryo-scanning electron microscopy

Scanning electron microscopy (SEM) was carried out using a Zeiss Supra 55 VP FEG SEM, fitted with a Deben CoolStage and attached to a Gatan Alto 2500 cryo system. A small drop of emulsion was pipetted into a brass rivet, which had been screwed into a sample holder connected to the transfer rod. The sample holder was immediately plunge frozen into liquid nitrogen before being loaded into the cryo preparation chamber of the SEM. The top of the emulsion drop was fractured followed by a short sublimation to remove surface frost. The sample was platinum coated inside the preparation chamber and then transferred onto the cooled stage inside the microscope for imaging.

### Animals

Intestinal samples from C57Bl/6 mice were collected following Schedule One approved euthanasia. All procedures were conducted in accordance with and approval of the University of East Anglia Animal Ethics Committees and covered by the appropriate licences under the UK Home Office Animal Procedures Act, 1986.

### Mucosal transport studies

10 cm sections of distal small intestine were flushed and collected into ice cold Ringer's solution (120 mM NaCl, 3 mM KCl, 0.5 mM MgCl, 1.25 mM CaCl and 23 mM NaHCO_3_ at pH 7.4) containing 10 mM Glucose. The most distal 1 cm was discarded to avoid the lymphoid follicle, the remaining tissue was cut into 1.5 cm sections and the serosa and muscularis layers were stripped off using fine forceps. Samples were mounted in low volume Ussing chambers (Physiologic Instruments, San Diego, USA) using P2404 slider inserts (exposed tissue area = 0.25 cm^2^). Both apical and basolateral compartments were filled with 1 mL Ringer's solution containing 10 mM Glucose in the basolateral solution and osmotically balanced with 10 mM mannitol in the apical solution. Samples were maintained at 37 °C and buffers were continually oxygenated and circulated using carbogen (5% CO_2_/95% O_2_). Open circuit potential difference (PD) was monitored continuously using a DVC1000 amplified (World Precision Instruments, New Haven, USA) and Ag–AgCl electrodes and 150 mM NaCl salt bridges. At 10 min intervals, a short circuit current (*I*_sc_) was applied to zero the PD. These data were collected using Spike2 v8.0 software (Cambridge Electronic Design, Cambridge UK) and used to calculate trans-mucosal resistance using Ohm's law. This serves as an indicator of barrier integrity throughout the experimental period. Similarly, following completion of the experiment, 10 μM forskolin and 100 μM 3-isobutyl-1-methylxanthine were added bi-laterally. Tissue segments that recorded a PD increase <1 mV were excluded from further analysis.^18^ Tissue segments were equilibrated for 30 min prior to commencement of the experimental period. At time 0, Ringer's solutions were replaced with 750 μL Ringer's + 250 μL digested emulsion on the apical side (+10 mM mannitol). On the basolateral side, the buffer was replaced with 750 μL Ringers + 250 μL simulated intestinal salt solution from the *in vitro* digestion protocol (+10 mM glucose) in order to minimise osmotic stress. Samples (100 μL) of the apical and basolateral solutions were collected at 0, 30, 60 and 90 min for determination of bile acid concentrations and the final buffer solutions were collected for assessment of free fatty acid transport. Tissue segments were collected for morphometric analysis.

### Bile acid analysis

The concentration of total bile acids in intestinal mucus samples was performed using the bile acids (enzymatic cycling) test kit (Alpha laboratories, Hampshire, UK) after centrifugation (16 000*g*, 5 min, 1 °C) and analysed as per the kit manufacturers protocol except the volumes of sample and reagents were reduced to perform the assay in a micro titre plate format. Mucus was mixed with four volumes of PBS then centrifuged (16 000*g*, 30 min, 1 °C). The absorbance was measured at 405 nm using a Bio-Rad Benchmark Plus microtitre plate spectrometer (Bio-Rad, Hertfordshire, UK) operated in the kinetic mode.

### Fatty acid analysis

Samples of digesta (0.5 ml) were evaporated to dryness in a Genevak EZ2 mk 2 evaporator (Genevac, UK) at 40 °C before being extracted with 0.5 ml of chloroform containing a mixture of internal standards comprising free fatty acid, monoglyceride, diglyceride and triglyceride (FFA heptadecanoic acid, MG monotridecanoin, DG dinonadecanoin, TG tripentadecanoin, (Nu-Chek Prep, USA).

All solid phase extraction steps were performed with flow under gravity except activation and brief drying steps. The chloroform extract was applied to a Bond Elut solid phase extraction cartridge (amino propyl, 500 mg, Agilent UK) immediately previously activated with 5 ml of hexane and very briefly dried under vacuum before sample application. The cartridge was eluted with 2 × 1 mL of chloroform : isopropanol (2 : 1 v/v) under gravity using the mixture to rinse out the sample tube and to wash the walls of the SPE cartridge to ensure complete loading of the sample. The column was eluted with a further 2 mL of chloroform : isopropanol (2 : 1 v/v), these fractions were combined and contained the neutral lipids. The cartridge was then eluted with 4 mL of chloroform : methanol : acetic acid (100 : 2 : 2 v/v), this fraction contained the free fatty acids. The solution of neutral lipids was evaporated to dryness at 40 °C (Genevac) and dissolved in 0.5 ml of hexane while warming at 40–55 °C in a water bath to ensure complete dissolution of any lipid crystals formed during the evaporation. The mixture was applied to a second amino propyl cartridge activated as before. A further 0.5 mL of hexane was used to dissolve any remaining lipid and wash the walls of the SPE cartridge to ensure complete loading before it was developed with a further 3.0 mL of hexane followed by 4.0 mL of hexane : dichloromethane : chloroform (88 : 10 : 2 v/v), these extracts were combined and contained the triglyceride components. The cartridge was washed with 4.0 mL of hexane : ethyl acetate (95 : 5 v/v) followed by 4.0 mL of hexane : ethyl acetate (85 : 15 v/v) and the washings discarded. The cartridge was eluted with 4.0 mL of hexane : ethyl acetate (20 : 80 v/v), this fraction contained the diglyceride components. The monoglyceride components were then eluted with 4.0 mL of chloroform : methanol (2 : 1 v/v). After evaporation to dryness at 40 °C (Genevac), the lipid fractions were dissolved in 0.5 ml of toluene (containing 0.1 mg ml^–1^ butylated hydroxytoluene, Sigma) in screw-capped glass tubes equipped with Teflon lined caps. Methanol (1.0 ml containing 2% sulphuric acid (Sigma)) was added and the tubes were flushed with nitrogen gas before being incubated overnight at 50 °C in a shaking incubator at 150 rpm (Incu-Shake Max, SciQuip, UK). Following cooling the solution was neutralised by the addition of 1.0 ml of 0.25 M potassium bicarbonate, 0.5 M potassium carbonate solution and mixed for 30 seconds. The mixture was extracted with 1.0 ml of hexane and following centrifugation (100*g*, one minute) the supernatant was collected and evaporated to dryness before being redissolved in 100 μl hexane and transferred to a vial for subsequent analysis.

FAMES were analysed on a Varian CP 3800 GC equipped with a split-splitless injector and a Saturn 2000 single quad MS detector (Agilent, UK) fitted with a 100 m SP2560 column, 0.25 mm ID, 0.2 μm film thickness (Sigma, UK). 1.0 μL of sample was injected, the injector was held at 240 °C, splitless for 1.5 minutes then split at 50 : 1 for 4.5 minutes before the split was reduced to 10 : 1 for the remainder of the separation. The column oven was held at 50 °C for 2 minutes before ramping to 150 °C at 100 °C per minute, held for one minute, ramped to 240 °C at 4 °C per minute before being held at this temperature for the remainder of the separation. Response factors were determined by analysing a gravimetric FAME standard (Supelco 37 Food FAME Mix (Sigma)).

### Statistics

Statistical analysis was performed using Graphpad Prism version 5.0 computer software (http://www.graghpad.com). Comparisons between two groups were made using a student's *t*-test and three groups (and their interactions) were evaluated using two-way ANOVAs and significance (*P* < 0.05) determined multiple comparison tests.

## Results

**Fig. 2:**
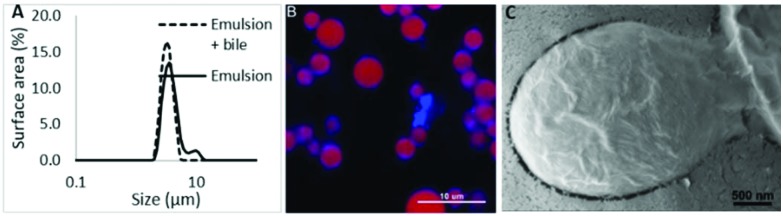
(A) The surface area weighted size distribution of the CNC stabilised emulsion in the absence and presence of bile. (B) A confocal image of the CNC stabilised emulsion showing the lipid droplets in red surrounded by a layer of CNCs in blue. (C) A cryo-SEM image of the stabilised emulsion showing the CNCs on the surface of a lipid droplet.

The aim of this study was to determine the fate of cellulose nanocrystals when used as Pickering stabilisers for a triglyceride emulsion and then after simulated oral consumption. With this aim in mind an emulsion was produced using sonication as described above. The size distribution of the emulsion is given in Fig. 2A. It is clear from the bimodal nature of the distribution, that the initial emulsion was slightly flocculated. However, the emulsion became well dispersed upon the addition of bile as shown by the narrow size distribution in Fig. 2A. The mean size of the emulsion was found to be unchanged after 3 days, demonstrating that the CNCs had formed an effective steric barrier on the emulsion droplets. Distribution of the CNCs on the emulsion surface was assessed microscopically. Confocal images, showing cross-sections of the stabilised emulsion shows a complete, but non-uniform distribution of CNCs on the surface (Fig. 2B), additionally, areas with higher CNC concentration appear to provide points for flocculation. Further assessment of the surface distribution was carried out using freeze fracture cryo-SEM (Fig. 2C) confirming the results seen by confocal microscopy, while the entire surface of the emulsion droplet is coated in CNCs, areas with higher density were observed. Additionally, the size and structure of the CNCs observed correlate with the data obtained by atomic force microscopy (Fig. 1).

**Fig. 3:**
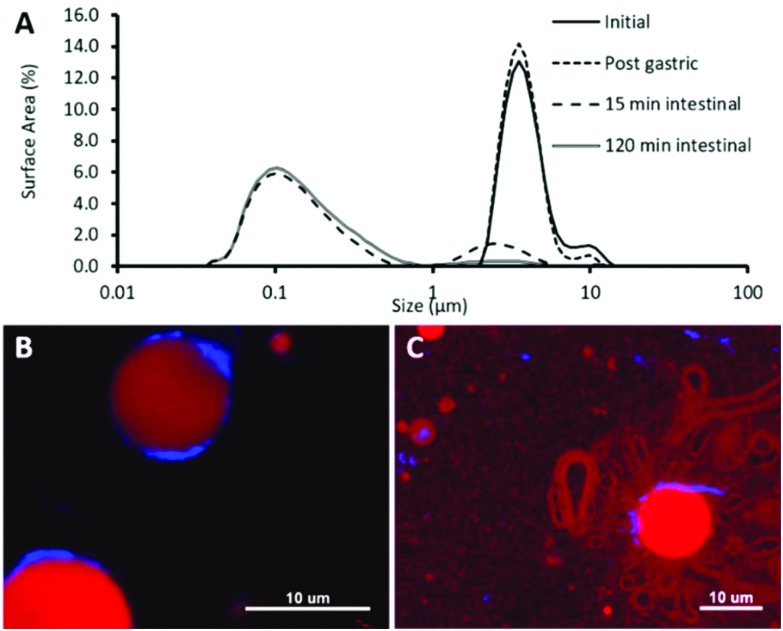
The surface area weighted size distribution of the CNC emulsion through digestion. The confocal images are of samples taken after the gastric (B) and intestinal (C) phases of digestion.

Having produced a stable emulsion, it was then exposed to simulated gastrointestinal digestion based on the standard Infogest conditions.^17^ The initial stage of digestion was 2 hours of gastric simulation at pH 3. Under these conditions, the CNC emulsion was less flocculated than the initial emulsion as shown in Fig. 3A and droplets size was unchanged. The confocal micrograph in Fig. 3B shows the emulsion droplets surrounded by a heterogeneous layer of CNCs demonstrating the propensity of the CNCs to aggregate, even in the surface layer of the droplets. In contrast, there was a significant, and rapid decrease in droplet size once the emulsion was passed into the intestinal phase of digestion. Prolonged exposure to the intestinal conditions digested essentially all the triglyceride emulsion leaving only particles of a few hundred nanometres. This is confirmed from the image in Fig. 3C, showing that the lipid droplets were no longer surrounded by a shell of CNCs but were instead surrounded by a variety of self-assembled structures formed from lipid hydrolysis products, primarily fatty acids and monoglycerides. The CNCs that are visible in the image are clearly in aggregates that are no longer associated with the lipid droplets. Indeed, some of the CNCs associated with the 10 μm droplet in the bottom right of the image can be seen in the process of being removed from the droplet interface as a coherent sheet. The image also highlights the propensity of the lipid hydrolysis products to form lamella structures.

**Fig. 4:**
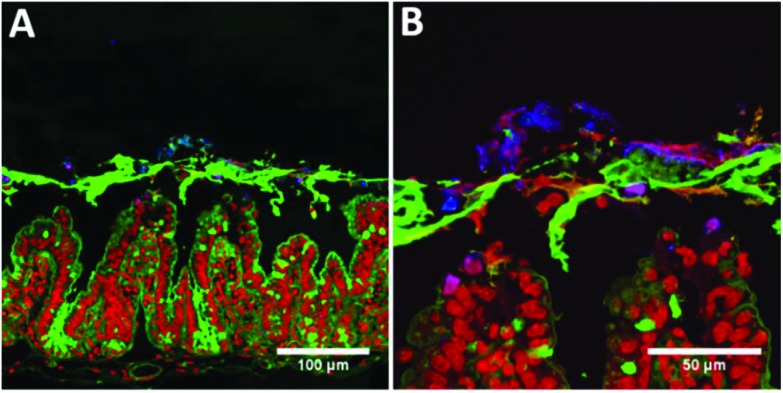
Confocal micrographs of intestinal mucosal segments following 120 min incubation in Ussing chambers. (A) 20× magnification, and (B) 63× magnification. Samples were stained with Calcofluor for CNCs (blue), wheatgerm agglutinin for mucins (green) and ToPro-3 for DNA (red).

In order to determine the fate of the CNCs once exposed to the intestinal mucosa, Ussing chambers containing murine intestinal mucosa had the apical chamber filled with a four-fold diluted sample of control or CNC emulsion following *in vitro* digestion. The images in Fig. 4 show the intestinal mucosa after 90 minutes exposure to CNCs. It is clear that the CNCs were trapped in the mucus layer, as there is no evidence of calcofluor staining in the mucosal tissue (Fig. 4A). Indeed, at higher magnification, there is little evidence that the CNCs were able to penetrate the mucus layer to any extent (Fig. 4B).

**Fig. 5:**
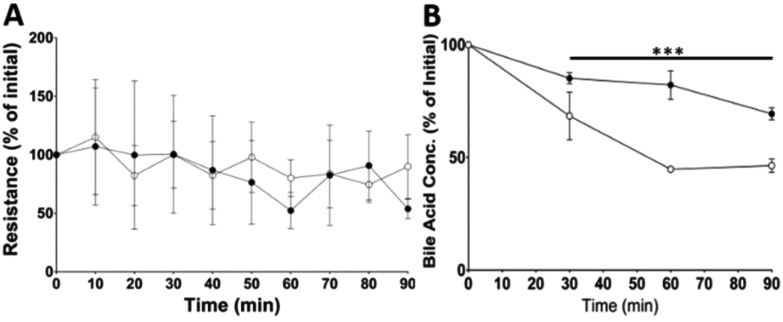
(A) Trans-mucosal resistance, (B) bile acid absorption in the distal ileum as measured by the decrease in apical bile concentration. Data for control (○) and CNCs ([black circle]) are expressed as a percentage change from baseline. *N* = 4–5 per group (***p < 0.001, by 2-way Annova).

In addition to determining the final location of the CNCs, the impact of the CNCs on the transport and absorption of bile acids (BA) and free fatty acids (FFAs) was also measured. This was achieved by measuring concentrations in both the apical and basolateral chambers as a function of time following *in vitro* digestion of the control and CNC stabilised emulsions. Epithelial barrier integrity was maintained throughout the experimental period as demonstrated trans-mucosal resistance (Fig. 5A) and no significant differences were observed between control and CNC groups. Fig. 5B shows bile absorption by ileal segments as the decrease in concentration over the length of the experiment. By 30 min, significantly less bile acid had been absorbed in samples containing the digested CNC stabilised emulsion compared to the control. No difference was observed in the basolateral buffer samples. This is most likely because values were at or below the limit of detection (data not shown) and any bile remaining in the tissue samples was not measured.

**Fig. 6:**
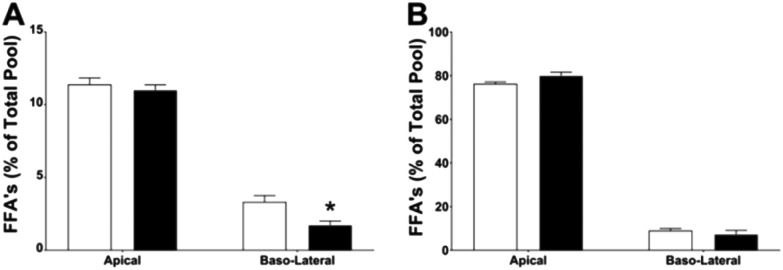
(A) Saturated and (B) unsaturated free fatty acid concentrations as a percentage of total free fatty acids in the apical and basolateral media of distal ileal explants after 90 min. Data for control (○) and CNCs ([black circle]) are expressed as a percentage change from baseline. *N* = 4–5 per group (***p < 0.05, by 2-way Annova).

The amount of individual fatty acids present as a proportion of the total fatty acid pool is outlined in Table 1. Overall, there was no significant difference in the total FFAs between the control and CNC stabilised emulsions even though a trend toward lower absorption was observed in Fig. 6B. Individually, Linoleic acid (C18:2) showed a significant proportion remaining on the apical side of the tissue after 90 minutes with the control being more than 10% lower. While this fatty acid made up the highest proportion of the total FFA profile, little was absorbed across the intestinal barrier and although there were differences in the basolateral proportions observed, these were not significant due to the low concentrations involved. However, significantly less stearic acid (C18:0) and eicosanoic acid (C20:0) were absorbed from the CNC emulsion following the 90 min incubation. This result suggests that CNC stabilisation of the emulsions may differentially affect absorption of saturated (Fig. 6A) and unsaturated (Fig. 6B) FFAs with inhibition of the absorption of saturated fatty acids.

**Table 1:** Absorption of free fatty acids across murine distal ileum in Ussing chambers. Data presented as a percentage of the total free fatty acid pool (*n* = 4–5 per group)

	Apical					Basolateral				
	Control		CNC		*p*-Value	Control		CNC		*p*-Value
	Mean	SEM	Mean	SEM		Mean	SEM	Mean	SEM	
C16:0	6.856	0.268	6.694	0.229	0.328	1.475	0.275	0.884	0.186	0.11
C16:1	0.504	0.072	0.591	0.176	0.346	0.182	0.116	0.281	0.134	0.608
C18:0	4.152	0.185	4.048	0.145	0.332	0.879	0.158	0.411	0.069	**0.022**
C18:1	30.89	1.351	29.21	0.983	0.169	7.924	0.729	6.224	1.616	0.41
C18:2	44.69	1.062	50.02	1.753	**0.023**	0.931	0.285	0.769	0.361	0.667
C20:0	0.334	0.088	0.258	0.067	0.253	0.966	0.061	0.435	0.128	**0.011**
Total	87.426	1.219	90.821	1.917	0.105	12.357	1.219	9.004	1.917	0.211

## Discussion

Initial characterisation of the CNCs using DLS and AFM has provided detail of their structure. This is consistent with previous electron microscopy studies,^19^,^20^ and values compare well with previous measurements from small angle neutron scattering in suspension that gave average dimensions of 195 ± 35 nm in length, 22 ± 3 nm in width, and 6 ± 0.2 nm in thickness.^21^ Additionally, this study builds on previous descriptions of the CNCs at the interface of a Pickering stabilised emulsion and the first in a food-ready system. The confocal imaging (including 3D, *z*-stack reconstructions, see ESI†) and the cryo-SEM demonstrate similar structure to those observed previously using styrene-in-water emulsions^16^ with uneven distribution on the oil-droplets surface. While it is unclear if the CNC aggregation occurred prior to, or during adhesion to the oil-droplets, it resulted in flocculation of the initial stabilised emulsion. This was most likely driven by a bridging mechanism.^10^

The surface area weighted size distribution and confocal imaging demonstrates that the CNC stabilised emulsions resist digestion through the gastric phase. In contrast to more conventional protein stabilised emulsions,^22^ CNC stabilised emulsions showed reduced flocculation in gastric conditions although this is unlikely due to changes in the electrostatic interactions as the charge is unlikely to have changed.^23^ However, the shift in particle size following the addition of pancreatin (which includes lipase) in the intestinal phase suggests the lipid within the emulsion was readily available for hydrolysis. Thus, there were some areas of the lipid droplets that were not protected by a CNC coating, allowing bile and more importantly, the lipase/co-lipase complex to access the oil/water interface. This is consistent with the 3D confocal reconstructions demonstrating areas of no CNC coverage and previous studies that suggest 40% coverage with un-sulphated CNCs.^16^ However, it is at odds with previous studies using CNCs and whey protein that were able to retard lipolysis.^24^ Indeed, in this case, significant digestion has occurred by 15 min and at 120 min, virtually all triglycerides had been hydrolysed with the mean particle size dropping to 100 nm, likely comprising CNC aggregates and larger self-assembled structures (the primary peak in Fig. 3A), and mixed micelles. Both the size distribution and the micrographs have shown that the CNCs are likely to form discrete aggregates in the intestinal lumen that not associated with lipid droplets. These data suggest that CNCs are an effective material for stabilising lipid emulsions, allowing survival in a gastric environment but disassembly under small intestinal conditions. Thus allowing effective delivery of lipid and lipid soluble compounds. Subsequently, the fate of the CNCs in the gut lumen is as aggregated clusters of crystals with a size of *circa* 100 nm.

In order to interact with the gut wall, particles must pass through the intestinal mucus layer. Murine tissue explants were used to ascertain the fate of the CNCs after *in vitro* digestion and their influence on lipid and bile absorption in the small intestine. Ussing-type perfusion chambers were used to determine permeability across the intact intestinal mucosal barrier, including the mucus layer. This study provides the first clear evidence that CNCs become trapped in the intestinal mucus layer post-digestion and are unable to reach the underlying enterocytes. Thus the proposed routes of absorption of the CNCs envisaged by others,^25^ such as through M-cells, through enterocytes by passive diffusion, through enterocytes by transcytosis or through the paracellular route, seem not to be realised.

Recent evidence has demonstrated that dietary polysaccharides can alter cholesterol and lipid homeostasis, potentially through the sequestration of bile acids, inhibiting their recycling and decreasing permeability of the mucus layer, slowing diffusion of digestion products self-assembled into mixed micelles. The cholesterol-lowering potential of dietary fibre is currently thought to involve the sequestering of bile acids, either through a direct interaction or as a result of increased luminal viscosity and entrapment of mixed micelles. Recent evidence demonstrates inhibition of fat digestion *in vitro*,^26^ potentially through interactions between water-soluble dietary fibre and pancreatic lipase. While soluble cellulose esters have demonstrated significant bile acid binding *in vitro*,^27^ less is known about the functional properties of the insoluble fibres, such as cellulose, within the gut environment. While this study demonstrates a decrease in bile absorption in tissue explants from the murine ileum, the mechanism and potential interaction with intestinal mucus remain unclear.

The hydrolysis of dietary fat is extremely efficient in the mammalian small intestine, but the range of digestion products is extremely diverse.^28^ However, all of the products readily self-assemble into structures of different size and only the smallest can diffuse through the mucus layer and be absorbed. While the mean micelle radius of sunflower oil following *in vitro* digestion is 3.05 nm ± 0.05 nm (ref. 29) they can also vary in size depending on the concentrations of saturated and unsaturated free fatty acids, bile salts, cholesterol, *etc.* Indeed, decreasing bile concentrations significantly increases micelle size^30^ and this will affect diffusion through the mucus layer.

Although saturated fatty acids only account for approximately 11% of the total fatty acid pool in this study, the data provides the first evidence that an insoluble dietary fibre may differentially regulate absorption of saturated and unsaturated free fatty acids in the small intestine, although further studies will be required to validate this finding. Early studies investigating lipid digestion demonstrated slower absorption of saturated compared to unsaturated fatty acids.^31^ The selective absorption may relate to the differences observed in bile acid uptake and/or the size of the mixed micelles formed during digestion through altered micelle packaging. Alternatively, several studies have demonstrated interactions between several types of dietary fibre and the mucus layer.^32^–^36^ It is proposed that the fibre could block the pores in the mucus network altering mucus permeability and reducing lipid micelle diffusion. The micrographs (Fig. 4) demonstrate that CNCs become trapped by the mucus layer and this may reduce its permeability to mixed micelles thereby reducing diffusion in a similar way to soluble fibres.^37^,^38^ If so, the fibre decreases the effective pore size of the mucus and limits diffusion of particulate species. These include mixed micelles, which are self-assembled structures of ≥10 nm and so are most likely to be affected. The mass transfer of other macronutrients such as peptides and reducing sugars are less likely to be influenced.

## Conclusions

The use of insoluble fibres, such a cellulose nanocrystal, may have multiple benefits over emulsifiers currently used in the food and pharmaceutical industries. The observed stability, low flocculation and coalescence in simulated gastric conditions will impact rates of lipolysis in the small intestine, potentially providing an effective delivery vehicle for lipophilic drugs. Importantly, this study demonstrated that the intestinal mucus layer prevents the CNCs from reaching the epithelium and that may modulate lipid absorption and bile recycling that could potentially lower plasma cholesterol. Thus, CNCs represent a safe and effective emulsifier. Determining the extent of these benefits would require further work required.

## Conflicts of interest

There are no conflicts to declare.

## Supplementary Material

Supplementary movie

Supplementary movie

Supplementary movie
